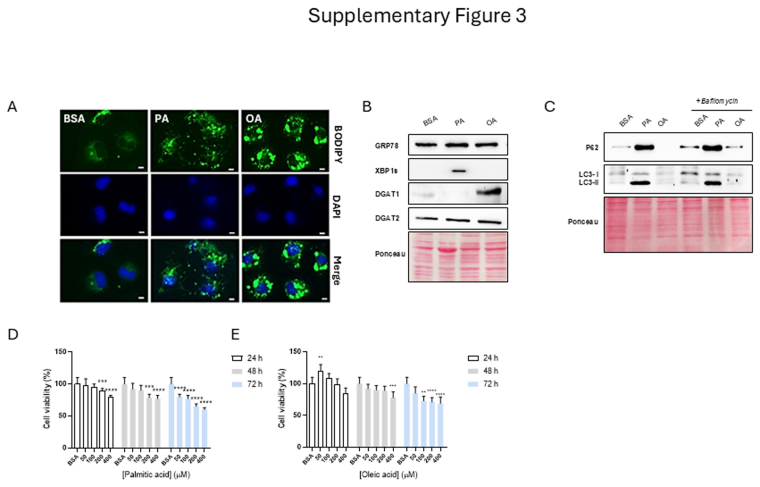# Lipotoxicity of palmitic acid is associated with DGAT1 downregulation and abolished by PPARα activation in liver cells

**DOI:** 10.1016/j.jlr.2024.100692

**Published:** 2024-11-05

**Authors:** Camilla Moliterni, Francesco Vari, Emily Schifano, Stefano Tacconi, Eleonora Stanca, Marzia Friuli, Serena Longo, Maria Conte, Stefano Salvioli, Davide Gnocchi, Antonio Mazzocca, Daniela Uccelletti, Daniele Vergara, Luciana Dini, Anna Maria Giudetti

**Affiliations:** 1 Department of Biology and Biotechnology “C. Darwin”, Sapienza University of Rome, Rome, Italy; 2 Department of Biological and Environmental Sciences and Technologies, University of Salento, Lecce, Italy; 3 Department of Physiology and Pharmacology “V. Erspamer”, Sapienza University of Rome, Rome, Italy; 4 Department of Medical and Surgical Sciences, University of Bologna, Bologna, Italy; 5 IRCCS Azienda Ospedaliero-Universitaria di Bologna, Bologna, Italy; 6 Interdisciplinary Department of Medicine, University of Bari School of Medicine, Bari, Italy

**Keywords:** diacylglycerol acyltransferase, endoplasmic reticulum stress, hepatic cells, lipid droplets, lipotoxicity

## Abstract

Lipotoxicity refers to the harmful effects of excess fatty acids on metabolic health, and it can vary depending on the type of fatty acids involved. Saturated and unsaturated fatty acids exhibit distinct effects, though the precise mechanisms behind these differences remain unclear. Here, we investigated the lipotoxicity of palmitic acid (PA), a saturated fatty acid, compared with oleic acid (OA), a monounsaturated fatty acid, in the hepatic cell line HuH7. Our results demonstrated that PA, unlike OA, induces lipotoxicity, endoplasmic reticulum (ER) stress, and autophagy inhibition. Compared with OA, PA treatment leads to less lipid droplet (LD) accumulation and a significant reduction in the mRNA and protein level of diacylglycerol acyltransferase 1 (DGAT1), a key enzyme of triacylglycerol synthesis. Using modulators of ER stress and autophagy, we established that DGAT1 downregulation by PA is closely linked to these cellular pathways. Notably, the ER stress inhibitor 4-phenylbutyrate can suppress PA-induced DGAT1 downregulation. Furthermore, knockdown of DGAT1 by siRNA or with A922500, a specific DGAT1 inhibitor, resulted in cell death, even with OA. Both PA and OA increased the oxygen consumption rate; however, the increase associated with PA was only partially coupled to ATP synthesis. Importantly, treatment with GW7647 a specific PPARα agonist mitigated the lipotoxic effects of PA, restoring PA-induced ER stress, autophagy block, and DGAT1 suppression. In conclusion, our study highlights the crucial role of DGAT1 in PA-induced lipotoxicity, broadening the knowledge of the mechanisms underlying hepatic lipotoxicity and providing the basis for potential therapeutic interventions.

Excess dietary fatty acids are often linked to pathological conditions such as obesity, insulin resistance, type two diabetes mellitus, and cardiovascular diseases (1). Among factors contributing to these conditions, a key role is played by the overload of triacylglycerols (TAGs) in sites other than adipose tissue, where lipids generally should not accumulate (2). The main form of TAG storage in cells is lipid droplets (LD), dynamic organelles found in all cell types, from yeast to humans. Regardless of their morphology, LDs share a similar structural organization, consisting of a phospholipid monolayer surrounding a core filled with neutral lipids, primarily TAGs and sterol esters (3). Various proteins, including perilipins (PLIN), associate with the monolayer to regulate different aspects of LD dynamics, such as growth and degradation, and are thought to influence LD positioning within the cell (3). LDs are closely associated with the endoplasmic reticulum (ER) (3, 4) where a significant proportion of cellular TAG is synthesized via the glycerol 3-phosphate pathway, which involves the esterification of fatty acids to a glycerol 3-phosphate backbone (5, 6, 7, 8). The final and committed step of TAG synthesis is the esterification of fatty acyl-CoA, catalyzed by diacylglycerol acyltransferase (DGAT) 1 and DGAT2. These enzymes differ in their biochemical, cellular, and physiological functions: DGAT1 is exclusively localized in the ER (9, 10) while DGAT2 is found in the ER and around LDs (9, 10). They have non-redundant roles; DGAT1 preferentially deals with exogenous fatty acids, whereas DGAT2 is important for TAG synthesis with de novo lipogenesis-derived fatty acids. Both enzymes are considered potential therapeutic targets for various diseases (11).

The accumulation of lipids in the liver, known as steatosis, can lead to more severe pathological conditions, such as steatohepatitis and cirrhosis (12). Considering the liver’s essential role in regulating lipid homeostasis, alteration in hepatic metabolism can impact the health of the entire organism.

Evidence shows that the type of dietary fatty acids can differently influence liver function. While a diet high in saturated fatty acids induces toxicity and cell death, an excess of monounsaturated or polyunsaturated fatty acids, although causing steatosis, is typically not toxic (13). This has shifted the prevailing paradigm that lipid accumulation is always toxic; instead, TAG accumulation may act as a protective mechanism against the harmful effects of free fatty acids (FFA). The greater the FFAs incorporated into TAGs, the lower their associated toxicity (14). Exposing hepatocytes to excess long-chain saturated fatty acids (SFA) induces inflammation, inhibits insulin signaling, and promotes ER stress, ultimately leading to cell death (15, 16). In contrast, unsaturated fatty acids are not toxic at comparable concentrations and can protect cells from SFA-induced lipotoxicity (17, 18, 19). Both in vitro and in vivo studies have demonstrated that inactivating or downregulating stearoyl-CoA desaturase 1 (SCD1), the key enzyme responsible for introducing double bonds into long-chain SFA (20), increases lipotoxicity, underscoring the role of acyl chain unsaturation in modulating toxicity.

Despite this knowledge, the exact molecular mechanisms underlying these effects remain partially understood and often controversial. Therefore, elucidating the impact of different lipid species on lipotoxicity is essential to develop targeted therapies for liver diseases.

Saturated palmitic acid (PA) and monounsaturated fatty acid oleic acid (OA) are among the most prevalent fatty acids in diet and serum (21). Studies indicate that PA exerts a toxic effect on the liver, while OA is non-cytotoxic and can protect against PA-induced toxicity (22, 23). Additionally, the endogenous conversion of PA to OA can reduce hepatotoxicity by promoting incorporation into TAG (24). With this study, we explored the molecular mechanisms underlying SFA lipotoxicity in hepatic cells. We found that PA-induced lipotoxicity was associated with ER stress and autophagy block. Notably, PA treatment led to fewer and smaller LD accumulation than OA, indicating a reduced capacity for incorporating into TAG. PA significantly downregulated DGAT1 expression at both mRNA and protein levels, with a mechanism dependent on ER stress and autophagy. PPARα activation alleviated PA-associated lipotoxicity and restored DGAT1 expression. In the simple animal model *Caenorhabditis elegans*, PA reduced lifespan, induced ER stress, and led to the accumulation of LDs with distinct morphologies compared to OA. In conclusion, by highlighting the role of DGAT1 as a key regulator of saturated fat-associated lipotoxicity, this study could be useful in setting up new therapeutic approaches for high-fat diet-associated liver disease treatments.

## Material and methods

### Cell treatments and reagents

Human hepatocellular carcinoma cell lines Huh-7 and HepG2 were maintained in Dulbecco's Modified Eagle Medium (DMEM) low glucose with 10% fetal bovine serum (FBS), 100 U/ml penicillin, 100 μg/ml streptomycin, and 2 mM glutamine. Cells were cultured at 37°C with 5% partial pressure of CO_2_ in a humidified atmosphere. Oleic acid (OA, COD. 75090), palmitic acid (PA, COD. 506345), palmitoleic acid (C16:1, COD. 76169), stearic acid (C 18:0, COD. 85679), and bovine serum albumin (BSA), and fraction V fatty acid-free (COD. 03117057001) were purchased from Merck. Fatty acids were dissolved in fatty-acid-free BSA at a final molar ratio of ∼2:1 fatty acids/BSA, close to the value observed in human serum (25), and diluted to a proper final concentration in DMEM just before cell treatments. Thapsigargin (COD. sc-24017), Myriocin (COD. sc-201397), and Tunicamycin (COD. Sc3506a) were purchased from Santa Cruz. Bafilomycin (COD. 54645S) and LY294002 (COD. 9901) were from Cell Signaling. GW7647 (COD. HY-13861), A922500 (COD. HY- 10038), 4-phorbol butyrate (PBA, COD. HY-A0281), and Torin 1 (COD. HY13003) were from MedChemExpress. 2-tetradecyl glycidic acid (TDGA) was kindly provided by Dr M. Guzmán (School of Biology, Complutense University, 28,040). Cell viability was assayed on adherent cells by the crystal violet assay method exactly as reported in (26). Intracellular peroxide was detected using 2′,7′-dichlorofluorescein diacetate (DCFH-DA, Sigma-Aldrich) staining as reported in (27).

### DGAT1 silencing by siRNA transfection

For the siRNA transfection experiments, Huh-7 cells were seeded in plates at 60% confluence and were transfected with a pool of three different DGAT1 siRNAs or with negative control (CRH5722). The oligonucleotides were purchased from Cohesion Biosciences (London, United Kingdom). According to the manufacturer's instructions, the cells were transfected with 20 nM of siRNA using ScreenFect®siRNA (S-4001) (ScreenFect). After 48 h, cells were harvested for mRNA and protein extraction.

### *Caenorhabditis elegans* growth conditions and lifespan assay

Nematodes were cultured as reported in (28). Lifespan analysis was conducted at 16°C and death was recorded when the nematodes ceased responding to gentle touch with a platinum wire. For the fertility assay, synchronized N2 worms were incubated at 16°C on NGM plates seeded with various treatments to allow for embryo laying. Subsequently, individual animals were transferred onto a fresh plate daily, and the total number of progenies was counted using a Zeiss Axiovert 25 microscope. This process was continued until the mother worms ceased laying eggs.

### LDs staining and fluorescence analyses

Oil Red O (ORO) and BODIPY stain protocols were used for LD analysis. Following fatty acid treatments, HuH-7 cells were washed with PBS, fixed with 4% paraformaldehyde in PBS pH 7.4 for 20 min, at room temperature, and stained with 0.5% ORO solution in isopropanol for 1 h. After PBS washing, the LD number and area were quantified using FIJI software version 2.9.0/1.53. To enhance LD detection, Z-stack projection was used to combine images from different focal planes, ensuring that LDs across z-axes were captured. The resulting 12 bit images were converted to 8 bit, Gaussian blurred (sigma = 1), and a threshold of 25. The images were then converted to a binary mask, and adjacent objects were separated using the watershed function. The “analyze particle” function identified LDs in a single cell. For BODIPY staining, cells were seeded onto coverslips in 12-well plates at 60%–80% confluence. After treatment, cells were washed twice with PBS and incubated for 15 min at 37°C with BODIPY 493/503 at a dilution of 1:1000 in PBS from a 1 mg/ml stock solution. After that, coverslips were washed three times with PBS and used for rapid image acquisition. Images were captured with a Zeiss LSM900 confocal laser scanning microscope (Zeiss) equipped with a 63×/1.40 NA oil immersion objective.

In *C. elegans*, ORO staining was performed on 1-day adult worms treated with fatty acids from embryo hatching. Worms were collected and fixed in a 60% isopropanol for 10 min, and stained with a working ORO solution (0.5% ORO in isopropanol/water 3:2). Following incubation for 1 h at room temperature, worms were centrifuged, washed with M9 buffer, and visualized under an Axio Observer fluorescence microscope (Zeiss) equipped with Apotome 3. Worms were imaged at two magnifications: 10X (with a 100 μm scale bar) and 63X objectives (with a 10 μm scale bar). LD assessment was conducted using FIJI software version 2.9.0/1.53. In the case of BODIPY 493/503 staining animals were processed as described in (29).

### RNA extraction and RT-qPCR analyses

Total RNA was extracted from cell pellets with the EasyPure RNA kit (TransGen Biotech Co., Ltd). RNA quantification was performed using a NanoDrop One Spectrophotometer (Thermo Scientific). For cDNA synthesis HIScript III RT SuperMix for qPCR (+gDNA wiper) kit was used (Vazyme Biotech), following the manufacturer’s instructions. Gene expression analyses were performed through real-time PCR using iTaq Universal Sybr Green Supermix (Bio-Rad) run on the CFX Connect Real-time System (BioRad). A comparative analysis was performed. Glyceraldehyde-3-phosphate dehydrogenase (*GAPDH*) and *RPLP0* were evaluated as housekeeping genes, resulting in stable overall samples. *RPLP0* was chosen as the reference gene. Primer sequences are reported in supplemental Table S1.

For analyses in worms, RNA was extracted from 200 1-day-old wild-type adults using the miRNeasy Micro Kit (Qiagen). The Real-time analysis was conducted using the ICycler IQ Multicolor Real-Time Detection System (Bio-Rad), according to (29). Selective primers (200 nM) were utilized, and their sequences are provided in supplemental Table S1.

### Oxygen consumption rate (OCR) measurements

Oxygen consumption rate (OCR) was measured with a polarographic approach using a Clark-type oxygen electrode in a water-jacketed chamber (Hansatech Instruments, Norfolk, UK) (30). Briefly, after trypsinization, cells were resuspended in a “respiration” buffer (buffer A = 75 mM sucrose, 5 mM KH_2_PO_4_, 40 mM KCl, 0.5 mM EDTA, 3 mM MgCl_2_, 30 mM Tris-HCl, pH 7.4) and inserted inside the water-jacketed chamber (final volume 1 ml). For the determination of the “respiratory fingerprint,” cells were treated with oligomycin (2 μg/μl), CCCP (1 μM), antimycin A (15 nM), and rotenone (1 μM).

### Western blot analyses

Proteins were extracted from cells using RIPA lysis buffer (Cell Signaling #9806). Total protein levels were determined using the Bradford method (Bio-Rad Laboratories). After boiling for 5 min, proteins were loaded and separated by SDS-polyacrylamide gel electrophoresis. The samples were then transferred onto a nitrocellulose membrane (Bio-Rad Laboratories) and blocked at room temperature for 1 h using 5% (w/v) non-fat milk in TBS-Tris buffer (Tris-buffered saline (TBS) plus 0.5% (v/v) Tween-20, TTBS). The membranes were incubated with the following primary antibodies: Glucose regulatory protein 78 (GRP78) (76-E6) (Santa Cruz # sc-13539, rat 1:1000), microtubule-associated protein 1α/1β-light chain 3 (MAP LC3α/β (G-4) Santa Cruz #sc-398822, mouse 1:1000), P62 (SQSTM1/p62 (D-3) Santa Cruz #sc-28359, Mouse 1:1000), DGAT1 (Santa Cruz #sc-32861, rabbit 1:1000), DGAT2 (Novus Biologicals, #NBP1-71701, mouse 1:1000), SCD1 (Santa Cruz #sc-58420, Mouse 1:1000), Adipose Triglyceride Lipase (ATGL, Santa Cruz, sc-365278, mouse 1:1000), PERK (Santa Cruz sc377400, mouse 1:1000), spliced XBP1 (XBP1s, Cell Signaling, 40435s, rabbit 1:1000), Beclin1 (Cell Signaling, 3495s, rabbit 1:1000). After washing with TTBS, the blots were incubated with peroxidase-conjugated monoclonal secondary antibodies (Sigma-Aldrich) at 1:10.000 dilutions at room temperature for 1–2 h. The blots were then washed thoroughly in TTBS. Western blotting analyses were performed using the Amersham ECL Advance Western Blotting Detection Kit (GE Healthcare) and the ChemiDoc system (Bio-Rad) was used for chemiluminescence measurement.

### Fatty acid β-oxidation measurements

The rate of fatty acid oxidation (FAO) was determined as the formation of labeled CO_2_ (31). Briefly, Huh-7 cells were incubated at 37°C in the presence of 0.5 mM albumin-bound [1–^14^C]PA (0.1 Ci/mol). After 20 min, reactions were stopped by 0.3 ml of 2 M perchloric acid (reactions proceed at a linear rate up to 45 min). At the same time, 0.15 ml of benzethonium hydroxide (1 M in methanol) was injected into a center well containing a filter paper. Samples were allowed to equilibrate for an additional hour at 4°C, and the center well (with the CO_2_ fixed as bicarbonate) was transferred into vials for radioactivity counting. To evaluate the contribution of peroxisome to β-oxidation, experiments were conducted in the presence of 5 μM TDGA, an irreversible specific inhibitor of carnitine-palmitoyltransferase-1 (CPT1), a mitochondrial rate-limiting enzyme for long-chain FAO, as reported in (32). TDGA was added to cells 1h before measurements.

### Thin-layer chromatography (TLC) analysis of lipids

Total lipids were extracted using methyl-tert-butyl ether, as reported in (33). Lipids were loaded on silica gel plates for thin-layer chromatography (TLC) separation. Plates were developed with hexane/ethyl ether/acetic acid (70/30/1; v/v/v) for neutral lipid separation, with toluene/methanol (70/30; v/v) for sphingolipid separation. After development, plates were uniformly sprayed with 10% cupric sulfate in 8% aqueous phosphoric acid, allowed to dry for 10 min at room temperature, and then placed into a 145°C oven for 10 min, as reported in (34). The ChemiDoc system (Bio-Rad) was used to measure spot intensity. Different lipid species were identified by developing specific standards in the same experimental conditions.

### Statistical analysis

Results are expressed as means ± standard deviation (SD). Statistical differences were evaluated using GraphPad Prism version 8.3.0 for Windows. The comparison was made using one-way analysis of variance (ANOVA) and 2-way ANOVA for oxygraphy experiments. After Tukey post hoc analysis, differences between groups were considered statistically significant when *P* < 0.05.

## Results

### Lipotoxicity and lipid droplet accumulation

**Supplementary Figure S1:**
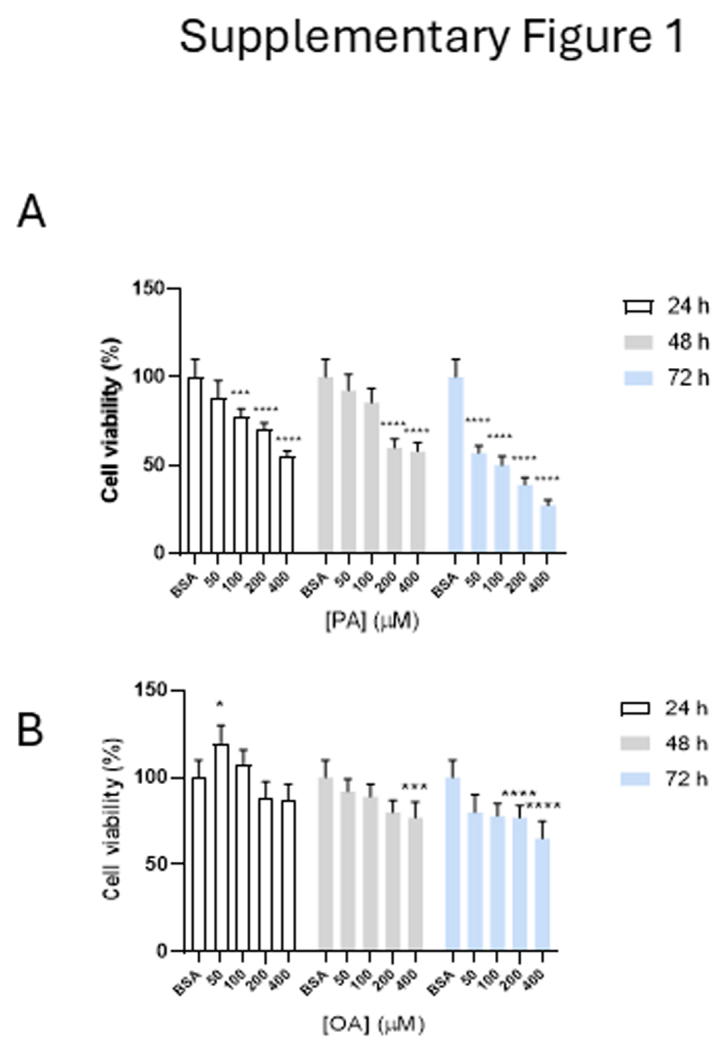


We first established a lipid accumulation model with OA or PA in liver cells to investigate the underlying lipotoxicity mechanism. Therefore, we conducted vitality assays in two different hepatic cell lines Huh-7 and HepG2. A dose- and time-dependent decrease in cell viability was measured after treatments with PA, compared to BSA and OA, in both cell lines, with a greater effect in Huh-7 cells (supplemental Figs. S1A, B and S3D, E). A slight but significant reduction in cell viability was observed by prolonging (>48 h) the incubation with maximal concentrations of OA (supplemental Figs. S1A, B and S3D, E).

**Fig. 1:**
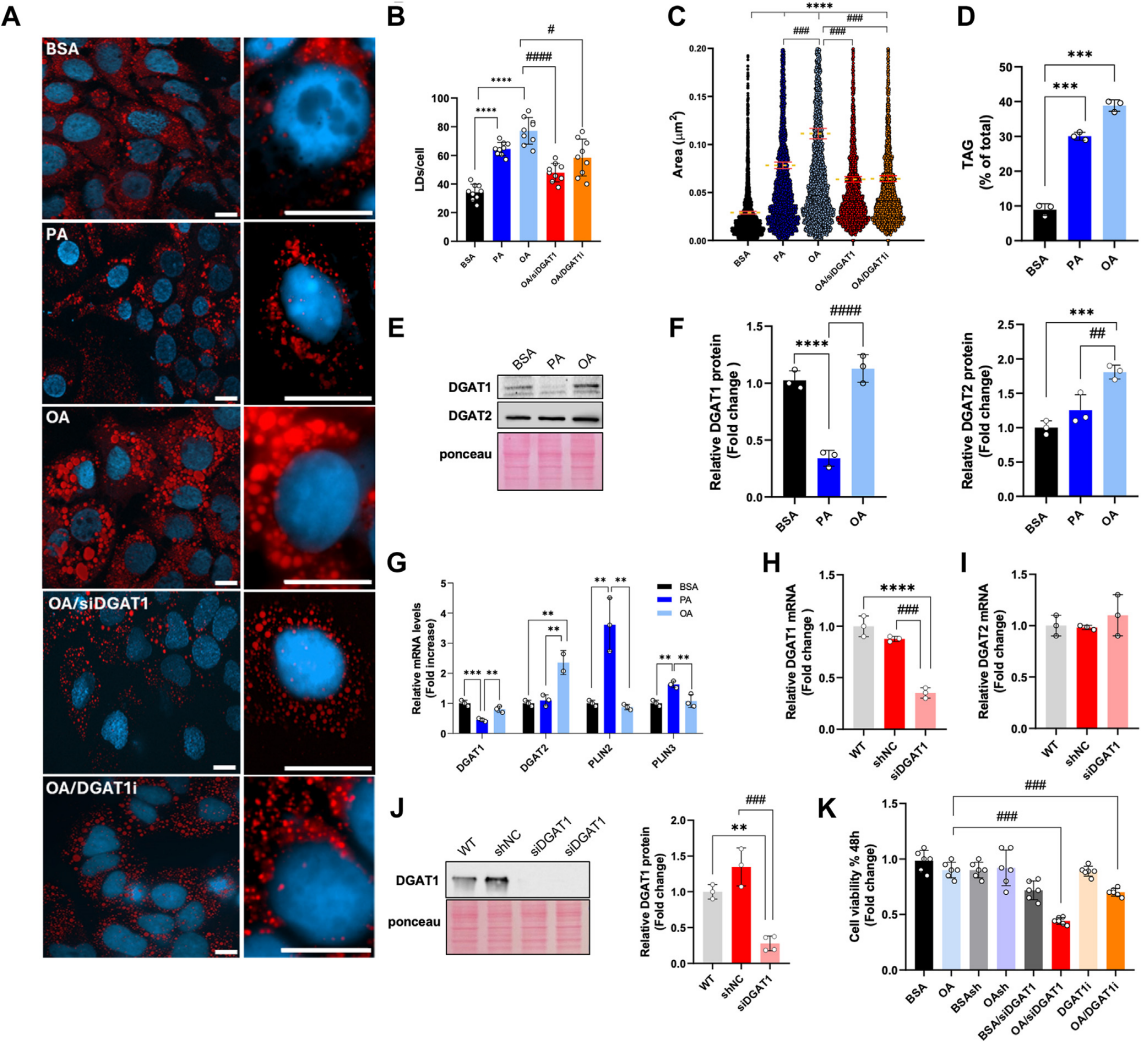
Analysis of LD and lipid metabolic enzymes in Huh-7 cells. A: Representative images obtained by confocal microscopy of Huh-7 cells stained with Oil Red O and DAPI after treatments for 24 h with BSA, 200 μM PA, or 200 μM OA, 200 μM OA + siRNADGAT1 (OA/siDGAT1), and 200 μM OA + 40 μM DGAT1 inhibitor (OA/DGAT1i). Image acquisition through Axio Observer fluorescence microscope (Zeiss) equipped with Apotome 3, magnification 40x air, and 63x oil (scale bar 10 μm). B, C: The number of LD/cells and area were obtained with FIJI software. Red bars represent the mean values of the area. (D) Triacylglycerol (TAG) quantification after TLC analysis. Values are expressed as % of total neutral lipids. (E, F) Representative Western blot, and relative quantifications, of DGAT1, and DGAT2 from Huh-7 cells treated with BSA, 200 μM PA, or 200 μM OA. (G) Real-time RT-PCR analysis of *DGAT1*, *DGAT2*, perilipin (*PLIN*) 2, and *PLIN3* in Huh-7 cells treated 24 h with BSA, 200 μM PA, or 200 μM OA. (H, I) Real-time RT-PCR and (J) Western blot analysis of DGAT1 in Huh-7 cells silenced for DGAT1 with siRNA. (K) Cell viability assay. Values are means ± SD. Asterisks indicate differences compared to the control, while hashtags denote group differences. (∗) *P* < 0.05; (∗∗) *P* < 0.01; (∗∗∗) *P* < 0.005; (∗∗∗∗) *P* < 0.001.

ORO staining and chromatographic analyses were used to follow the development of lipid accumulation. Compared to PA, OA induced a greater number of larger and brighter LDs in the central region of the cells. In contrast, PA was incorporated into smaller, less numerous, and evenly distributed LDs (Fig. 1A–C). TLC analysis confirmed significantly higher TAG accumulation upon equimolar administration of OA than PA (Fig. 1D).

**Supplementary Figure S2:**
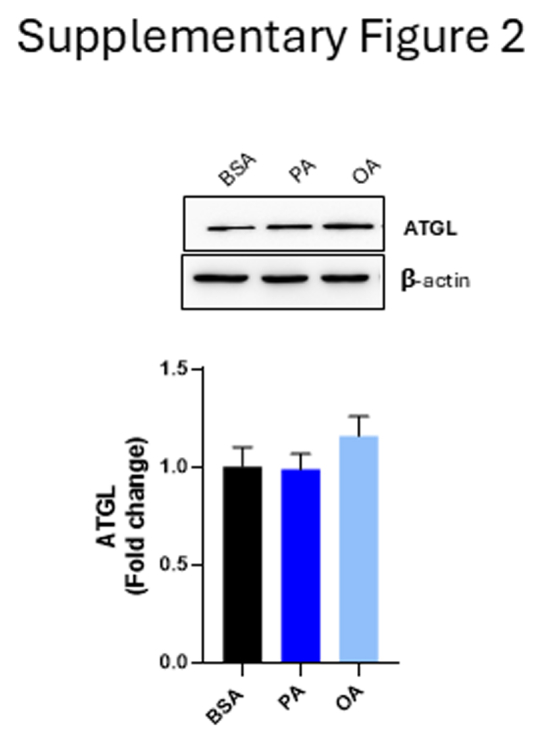


We analyzed the expression of key enzymes involved in LD metabolism. PA significantly reduced DGAT1 protein levels compared to OA and BSA (Fig. 1E, F), while OA markedly increased DGAT2 expression relative to the other groups (Fig. 1E, F). These changes were correlated with lower *DGAT1* mRNA levels in PA-treated cells compared to those treated with BSA and OA, and higher *DGAT2* mRNA levels in OA-treated cells compared to BSA and PA (Fig. 1G). No significant changes were observed in the levels of ATGL, an enzyme involved in the lipolysis of TAG into diacylglycerols and free fatty acids (supplemental Fig. S2). Additionally, mRNA levels of *PLIN2* and *PLIN3*, proteins closely associated with LDs and involved in their remodeling, were significantly elevated in PA-treated cells compared to BSA and OA (Fig. 1G).

Given the marked reduction of DGAT1 expression induced by PA, we investigated its potential role in lipotoxicity. We conducted experiments where OA was added to cells silenced for DGAT1 (siDGAT1) or treated with the specific DGAT1 inhibitor A922500 (DGAT1i). In the siDGAT1 cells, DGAT1 expression was drastically reduced at both mRNA and protein levels (Fig. 1H, L), without inducing compensatory effects on the DGAT2 isoform (Fig. 1I). Under these conditions, OA/siRNA cells accumulated fewer LDs than control cells (OAsh). At a concentration that diminished TAG synthesis by about 50% (data not shown), A922500 induced a reduction in LD accumulation in cells treated with OA (OA/DGAT1i) (Fig. 1A-C), In both scenarios, reduced cell viability was noted (Fig. 1M), compared with OA, mirroring the conditions observed in PA-treated cells.

### Palmitic acid-induced DGAT1 downregulation is related to ER stress and autophagy block

**Fig. 2:**
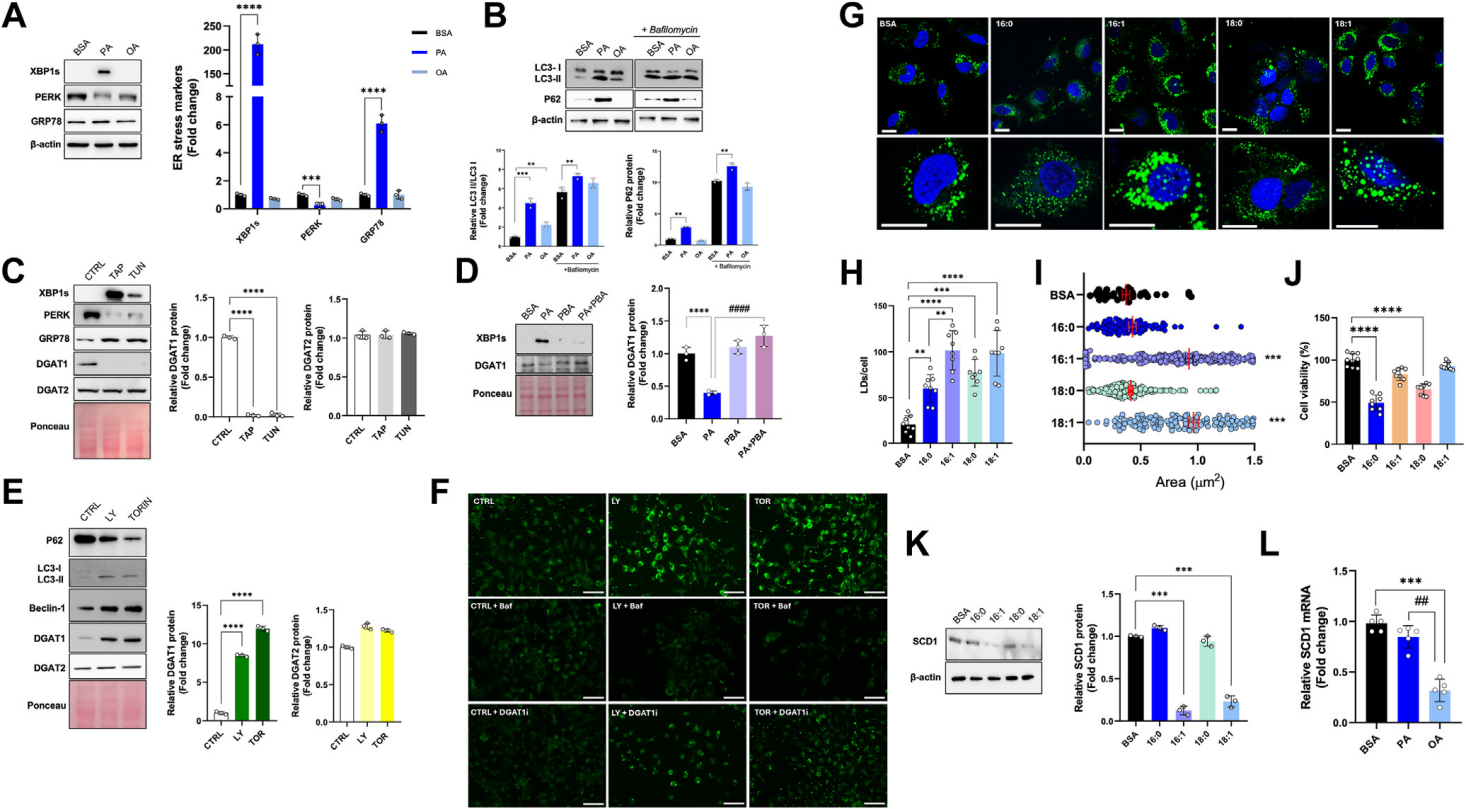
DGAT1 inhibition is related to endoplasmic reticulum stress and autophagy block and depends on fatty acyl-chain saturation. A: Western blot analysis, and relative quantification, of spliced XBP1 (XBP1s), PERK, and GRP78 in cells treated with BSA, 200 μM PA, or 200 μM OA. B: Western blot analysis and relative quantification of LC3I, LC3II, and P62 in cells treated with BSA, 200 μM PA, or 200 μM OA, in the presence or absence of 200 nM Bafilomycin added 2h before incubation ending. C: Western blot analysis, of XBP1s, PERK, GRP78, DGAT1, and DGAT2, and relative quantification of DGAT1 and DGAT2, in control (CTRL) and cells treated with 1 μM Thapsigargin and 1 μM Tunicamycin for 24 h. D: Western blot analysis of XBP1s and DGAT1, and relative quantification of DGAT1, in BSA cells and treated with 500 nM 4-phorbol butyrate (PBA) for 24 h. E: Western blot analysis of LC3I, LC3II, Beclin-1, DGAT1, and DGAT2, and relative quantification of DGAT1 and DGAT2 in CTRL and cells treated with 10 μM LY20094002 and 200 nM Torin 1. F: Images of BODIPY-stained Huh-7 neutral lipids, obtained by fluorescent microscopy after treatments with 10 μM LY20094002 and 200 nM Torin 1, in the presence or absence of 200 nM Bafilomycin and 40 μM DGAT1 inhibitor. (Scale bar 100 μm). G: Confocal microscopy after BODIPY and DAPI stains nuclei in Huh-7 cells treated for 24 h with BSA, 200 μM PA, 200 μM palmitoleic acid (C16:1), 200 μM stearic acid (C18:0), 200 μM OA. H, I: The measurements of numbers and area of LDs/cells were obtained with FIJI software. Red bars represent the mean values of the LD area. J: Vitality test for Huh-7 cells treated for 24 h with BSA, 200 μM PA, 200 μM C16:1, 200 μM C18:0, and 200 μM OA. K: Western blot analysis of stearoyl-CoA desaturase-1 (SCD1) and relative quantification in Huh-7 cells treated with BSA, 200 μM PA, 200 μM C16:1, 200 μM C18:0, and 200 μM OA. L: Real-time RT-PCR analysis of *SCD1* in Huh-7 cells treated for 24 h with BSA, 200 μM PA, or 200 μM OA. Asterisks indicate differences compared to the control, while hashtags among groups. Values are means ± SD. (∗) *P* < 0.05; (∗∗) *P* < 0.01; (∗∗∗) *P* < 0.005; (∗∗∗∗) *P* < 0.001.

ER stress and autophagy are closely associated with lipotoxicity, especially in the context of excess FFAs and disrupted lipid metabolism (16). We found that PA, compared to OA, induced ER stress, as indicated by increased expression of XBP1s and GRP78, along with decreased expression of PERK, which are specific ER stress markers (Fig. 2A). Additionally, PA significantly increased the LC3-II/LC3-I ratio and P62 expression (Fig. 2B). Treatment with bafilomycin 1, which targets the vacuole-type H + -ATPase preventing lysosomal acidification, does not increase the amount of autophagic dots promoted by PA treatment, confirming that PA inhibits the autophagic flux (Fig. 2B). Similar results were observed in the hepatoblastoma cell line HepG2, where PA, compared to OA, induced ER stress, an autophagic block, and a significant reduction in DGAT1 protein expression (see supplemental Fig. S3).

To explore the association between DGAT1 and ER stress we measured DGAT1 expression in the presence of Thapsigargin and tunicamycin, two specific ER stress activators. In treatments with Thapsigargin and tunicamycin, DGAT1 expression was significantly reduced (Fig. 2C). In the same context, DGAT2 expression did not change (Fig. 2C). Notably, the specific ER stress inhibitor PBA, at a concentration reducing PA-induced XBP1s, restored DGAT1 expression (Fig. 2D).

To further investigate whether the PA-induced reduction of DGAT1 was related to the block of autophagy flux, we used LY294002 and Torin 1, two autophagy activators. Both compounds increased DGAT1 but not DGAT2 expression (Fig. 2E). These findings suggest that DGAT1 suppression depends on ER stress and autophagy block.

Interestingly, LY294002 and Torin 1 promoted LD accumulation in HuH-7 cells, and the effect was completely prevented by bafilomycin and DGAT1i (Fig. 2F). These findings support the notion that DGAT1 plays a crucial role in LD formation associated with the autophagic process.

To assess the impact of fatty acid unsaturation on lipotoxicity, we treated cells with various fatty acids, including PA, OA, palmitoleic acid, and stearic acid. Analysis of BODIPY-stained LDs revealed morphological differences among the treatment groups (Fig. 2G). Quantitative analysis of LDs showed that PA and stearic acid resulted in fewer and smaller LDs than OA and palmitoleic acid (Fig. 2H, I). Notably, palmitoleic acid exhibited no significant cytotoxicity at the same concentration as PA, while stearic acid was cytotoxic, unlike OA (Fig. 2L). Since SCD1 is the key enzyme in fatty acid desaturation, we investigated the modulation of SCD1 by these fatty acids. Compared to BSA, the monounsaturated fatty acids palmitoleic acid and OA significantly reduced SCD1 protein expression (Fig. 2M). In contrast, saturated fatty acids did not alter SCD1 expression. Additionally, OA treatment led to a marked decrease in *SCD1* mRNA levels compared to PA (Fig. 2N).

### Palmitic and oleic acid are differently metabolized in hepatic cells

**Fig. 3:**
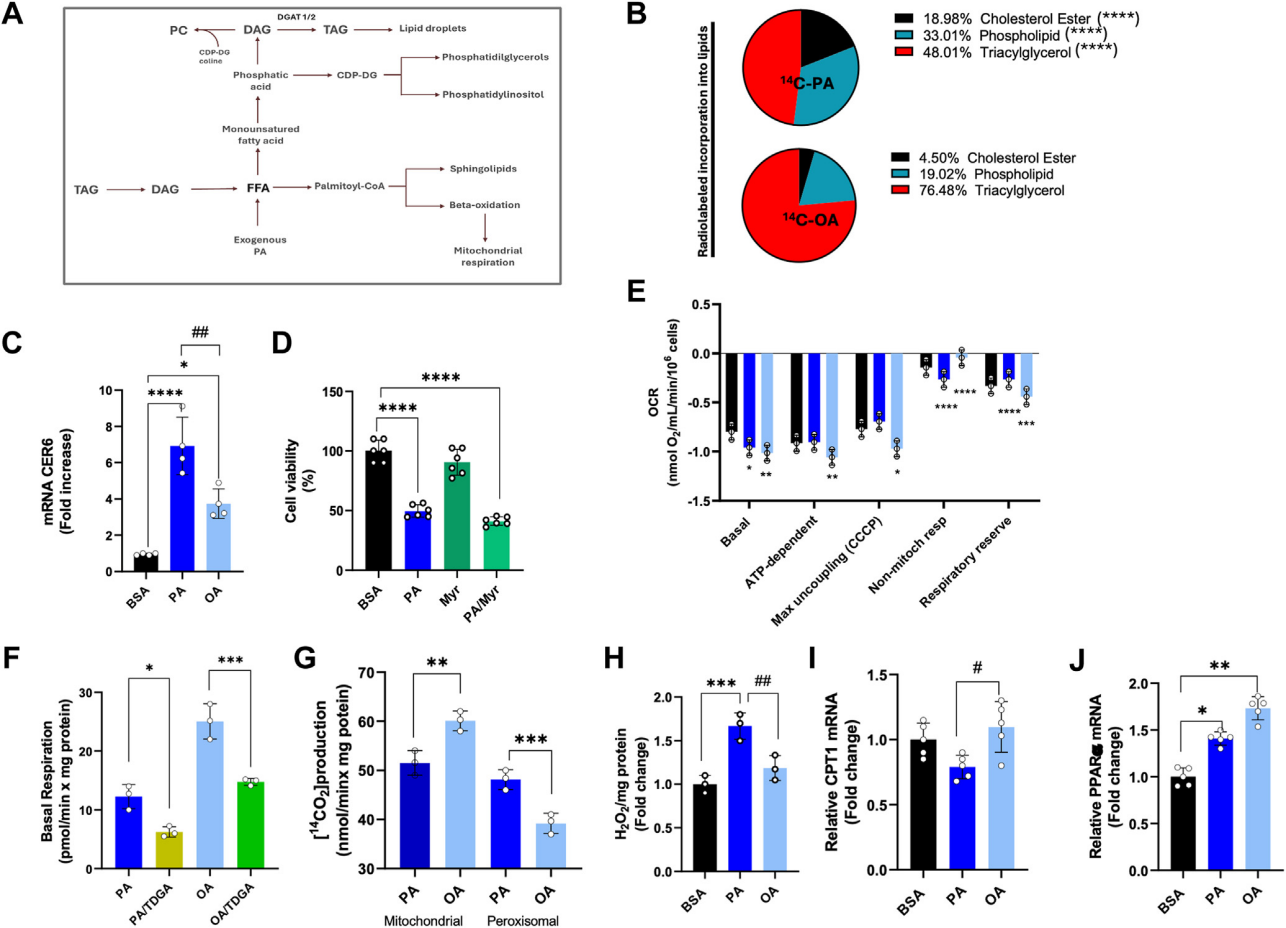
Lipid composition and bioenergetics parameters of Huh-7 cells. A: Schematic representation of the fate of exogenous added fatty acids (FFA) in cells. Once entered in the cells, FFA can be converted into esters of SH-CoA and incorporated into complex lipids or β-oxidized. B: Distribution of [1^-14^C]-PA or [1^-14^C]-OA in Huh-7 after 4 h of incubation with the labeled substrates. C: Real-time RT-PCR analysis of *CER6* in Huh-7 cells treated for 48 h with BSA, 200 μM PA, or 200 μM OA. D: Vitality test for Huh-7 cells treated for 24 h with BSA, 200 μM PA, 3.7 μM Myriocin (Myr), and PA + Myr. E: Basal respiration measurements in Huh-7 cells treated for 24 h with BSA, 200 μM PA, or 200 μM OA. Cells were inserted in the oxygraphic chamber and treated with 2 μg/μl oligomycin, 1 μM CCCP, 15 nM antimycin A, and 1 μM rotenone. *Basal* = basal cell respiration value; ATP-dependent = basal-oligomycin value (ATP-dep.); *Max uncoupling (CCCP)* = CCCP value (MaxUN); *non-mitochondrial respiration* = basal-CCCP value (Non-mit.); *Respiratory Reserve* = CCCP-antimycin A value (RR). Oligomycin: inhibitor of complex V of mitochondrial oxidative phosphorylation (ATP synthase). CCCP: carbonyl cyanide m-chlorophenylhydrazone, an uncoupler of mitochondrial oxidative phosphorylation; antimycin A: inhibitor of complex III of mitochondrial oxidative phosphorylation. Data are reported as nmol O_2_/ml/min/10^6^ cells. F: Basal respiration of Huh-7 cells treated for 24 h with BSA, 200 μM PA, or 200 μM OA, in the presence or absence of 5 μM 2-tetradecyl glycidic acid (TDGA). G: β-oxidation in Huh-7 cells treated with BSA, 200 μM PA, or 200 μM OA, in the presence of or absence of 5 μM TDGA. The picture reports the mitochondrial and peroxisomal contribution to the total β-oxidation. H: Hydrogen peroxide (H_2_O_2_) production from Huh-7 cells after 24 h with BSA, 200 μM PA, or 200 μM OA. (I, J) Real-time RT-PCR analysis of *CPT1* and *PPARα* in Huh-7 cells 24-hour-treated with BSA, 200 μM PA, or 200 μM OA. Asterisks indicate differences compared to the control, while hashtags among groups. Values are means ± SD. (∗) *P* < 0.05; (∗∗) *P* < 0.01; (∗∗∗) *P* < 0.005; (∗∗∗∗) *P* < 0.001.

Once internalized, fatty acids can be stored as TAG, converted into phospholipids and sphingolipids (such as ceramide and sphingomyelin), or undergo β-oxidation for ATP production (Fig. 3A). To investigate the fate of PA and OA in liver cells, we incubated the cells with [1–^14^C]PA (53 mCi/mmol) or [1–^14^C]OA (53 mCi/mmol) for 4 h, followed by TLC analysis of total lipids. Our results indicate that PA and OA are differently distributed in cell lipids. Specifically, PA is incorporated into cholesteryl esters and phospholipids to a greater extent than OA, while OA is more significantly incorporated into TAG compared to PA (Fig. 3B). PA is a substrate for the de novo synthesis of ceramide, a toxic lipid species (35). Although we found no significant differences in ceramide levels between PA- and OA-treated cells (data not shown), PA compared to OA-treated cells induced significantly higher mRNA expression of ceramide synthase 6 (*CERS6*), which synthesizes C16 acyl chain ceramides (Fig. 3C). Importantly, treatment with myriocin, an inhibitor of the key enzyme in de novo ceramide synthesis, did not alleviate PA-associated lipotoxicity (Fig. 3D), suggesting that this pathway is not involved in PA-induced lipotoxicity.

We evaluated the potential link between PA-induced lipotoxicity and changes in mitochondrial function by measuring the oxygen consumption rate (OCR). Compared to BSA, PA, and OA increased basal respiration, with OA exhibiting a more pronounced effect (Fig. 3E). The higher basal respiration observed in OA-treated cells was associated with enhanced ATP-dependent respiration. In contrast, the increase in OCR induced by PA was uncoupled from ATP synthesis, indicating reduced oxidative phosphorylation (OXPHOS) efficiency, whereas OA improved this efficiency. OA also resulted in lower non-mitochondrial respiration compared to the elevated level of PA-treated cells. Furthermore, OA increased the respiratory reserve (RR), which was diminished in PA-treated cells (Fig. 3E). When basal respiration was measured in the presence of TDGA, a specific irreversible inhibitor of CPT1, OA-induced basal respiration was greatly reduced than PA (Fig. 3F). These results suggest that β-oxidation from OA contributes more than PA to OCR. To differentiate the contributions of mitochondria and peroxisomes to β-oxidation, we measured the rate of labeled CO_2_ production in the presence of TDGA. The peroxisomal β-oxidation, determined as the difference between total and TDGA-sensitive β-oxidation, was significantly higher in PA-treated cells than in OA-treated cells (Fig. 3G) and correlated with increased levels of H_2_O_2_ (Fig. 3H). The observed differences in β-oxidation were not linked to changes in *CPT1* mRNA levels, which remained stable across treatments (Fig. 3I). However, we noted a small but significant increase in *PPARα* mRNA levels, a key regulator of fatty acid metabolism in the liver, particularly in OA-treated cells (Fig. 3L).

### PPARα activation reverses palmitic acid-induced lipotoxicity

**Fig. 4:**
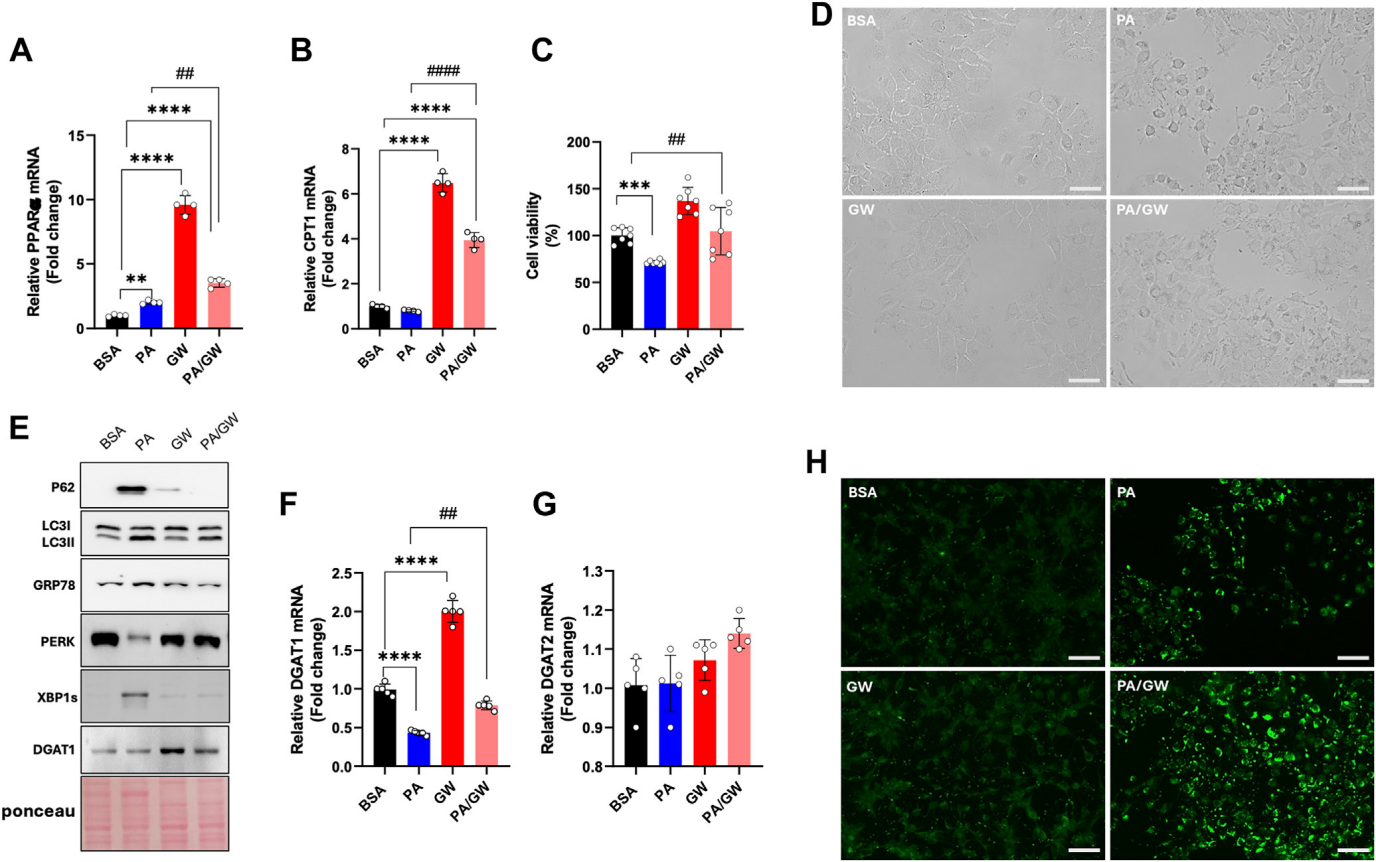
PPARα agonists rescue PA-induced lipotoxicity in hepatic cells. A, B: Real-time RT-PCR analysis of *PPARα* and *CPT1* in Huh-7 cells 24-hour-treated with BSA, 200 μM PA, 10 μM GW7647, 200 μM PA + 10 μM GW7647. C: Crystal violet vitality test in Huh-7 cells incubated for 24 h with BSA, 200 μM PA, 10 μM GW7647, 200 μM PA + 10 μM GW7647. D: Bright field microscopy images of HuH-7 cells treated with BSA, 200 μM PA, 10 μM GW7647, 200 μM PA + 10 μM GW7647. E: Western blot analysis of P62, LC3I, LC3II, GRP78, PERK, XBP1s, and DGAT1 in Huh-7 cells treated with BSA, 200 μM PA, 10 μM GW7647, 200 μM PA + 10 μM GW7647. F, G: Real-time RT-PCR analysis of *DGAT1* and *DGAT2* in Huh-7 cells treated for 24 h with BSA, 200 μM PA, 10 μM GW7647, 200 μM PA + 10 μM GW7647. H: Images of BODIPY-stained Huh-7 neutral lipids, obtained by fluorescent microscopy after cell treatments with BSA, 200 μM PA, 10 μM GW7647, 200 μM PA + 10 μM GW7647. (Scale bar 100 μm). Asterisks indicate differences compared to the control, while hashtags among groups. Values are means ± SD. (∗∗) *P* < 0.01; (∗∗∗) *P* < 0.005; (∗∗∗∗) *P* < 0.001.

PPARα agonists can mitigate hepatic fatty acid accumulation by stimulating β-oxidation (36). Additionally, PPARα can modulate DGAT expression (37). To investigate the role of PPARα in PA-inducing DGAT1 downregulation, we utilized the synthetic PPARα agonist GW7647. As expected, GW7647 significantly increased *PPARα* mRNA levels (Fig. 4A). Co-incubation with PA further enhanced PPARα expression beyond that observed in PA-treated cells (Fig. 4A). This was also reflected in *CPT1* mRNA levels, which were significantly elevated in the GW7647/PA combination compared to PA alone (Fig. 4B). Additionally, the PA/GW7647 combination significantly reduced PA-associated lipotoxicity (Fig. 4C) and improved cell morphology altered by PA treatment (Fig. 4D). Co-incubation with the PPARα activator notably decreased the PA-induced expression of ER stress and autophagy markers (Fig. 4E). GW7647 also upregulated DGAT1 at both the protein and mRNA levels compared to PA alone (Fig. 4E, F), while *DGAT2* mRNA expression remained largely unchanged (Fig. 4G). Interestingly, BODIPY-stained cells treated with the PA/GW7647 combination showed greater accumulation of green-stained droplets than those treated with PA alone (Fig. 4H).

### Metabolic alterations induced by palmitic acid in *Caenorhabditis elegans* mirror those seen in cultured liver cells

**Fig. 5:**
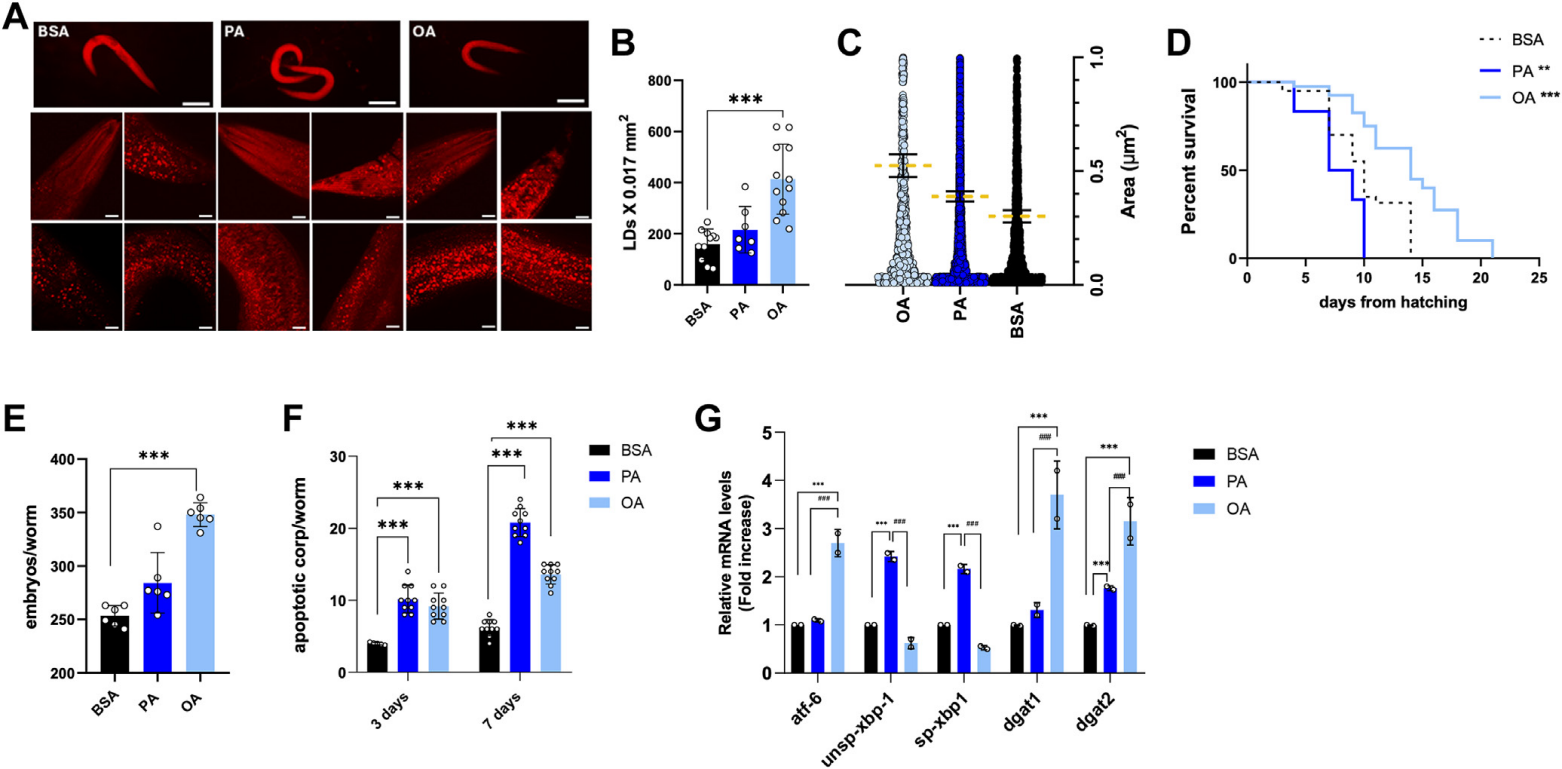
Impact of OA and PA on nematode life span, lipid metabolism enzymes, and ER stress. A: Oil Red O staining of 1-day adult worms treated with BSA, 200 μM OA, or 200 μM PA and (B-C) related LD number and area quantification. D: Kaplan-Meier survival plot of N2 worms fed with heat-killed OP50 and supplemented with BSA, 200 μM OA, or 200 μM PA. E: Average embryo production per worm. F: Apoptotic corpses in 3-day adults and 7-day adults supplemented with 200 μM OA or 200 μM PA compared to the BSA-treated control population. G: Real-time qPCR analysis of *Atf-6*, *unsp-Xbp-1*, *sp-Xbp-1, Dgat-1,* and *Dgat-2* genes in treated 1-day adults. Asterisks indicate the *P*-values (log-rank test) normalized to the control. Hashtags refer to differences among groups. (∗∗∗) *P* < 0.005; (∗∗∗∗) *P* < 0.001.

*Caenorhabditis elegans* is a valuable model organism for studying different human diseases at both metabolic and genomic levels in vivo. This nematode has been widely utilized across various research fields (27, 28, 38). Notably, its intestine retains many specialized liver functions (39) and key aspects of lipid metabolism are conserved between nematodes and mammals, with lipid synthesis and modification occurring primarily in the *Caenorhabditis elegans* intestine (40). We evaluated whether PA could replicate its effects in *Caenorhabditis elegans*. ORO-stained lipid droplets (LDs) displayed characteristics like those in liver cells, with OA inducing larger and more abundant LDs than PA (Fig. 5A–C). Viability analysis of the wild-type population indicated that PA significantly reduced nematode lifespan compared to BSA, with 50% viability reached by day 9 (Fig. 5D). In contrast, OA treatment extended lifespan, achieving 50% viability by day 14, while BSA control reached 50% viability by day 10 (Fig. 5D). Fertility analysis (Fig. 5E) showed a notable increase in offspring numbers with OA supplementation compared to BSA.

Examination of apoptotic bodies, quantified using fluorescence microscopy after acridine orange staining, revealed increased apoptosis following both PA and OA treatments at day 3 (Fig. 5F). This effect intensified by day 7, with PA treatment resulting in a greater accumulation of apoptotic bodies than OA (Fig. 5F).

We also analyzed the expression of genes associated with ER stress, including *Atf6* and the spliced and unspliced forms of *Xbp1* (Fig. 5G). While *Atf6* levels increased in OA-treated nematodes compared to controls and those treated with PA, both spliced and unspliced forms of *Xbp1* were significantly elevated in PA-treated nematodes compared to BSA- and OA-treated groups (Fig. 5G). Furthermore, mRNA levels of *Dgat1* and *Dgat2* were higher in OA-treated nematodes than in PA and BSA (Fig. 5G), whereas *Dgat1* levels did not change significantly in PA-treated nematodes compared to controls.

## Discussion

Lipotoxicity from chronic high exposure to FFAs poses a significant threat to cellular health and is implicated in various metabolic diseases. Understanding the underlying mechanisms is crucial for developing strategies to mitigate lipotoxicity and improve metabolic health.

The lipotoxicity of saturated fatty acids is linked to ER stress and impaired autophagic flux, both of which contribute to cellular dysfunction and metabolic diseases (41, 42, 43, 44, 45, 46). Our findings demonstrate that PA increases cell death, activates ER stress responses, and impairs autophagic function in both hepatic cell models and in the in vivo model, *Caenorhabditis elegans*. PA results in lower LD accumulation, compared to OA and downregulates DGAT1, potentially contributing to metabolic dysfunction. Previous studies have highlighted the crucial role of DGAT1 in TAG recycling and its protective effects against FFA-induced lipotoxicity (47). DGAT1 is essential for regulating TAG levels during adipocyte differentiation, thereby promoting proper lipid storage, and preventing lipotoxicity (48). Our study reveals, for the first time, how PA-induced ER stress and impaired autophagy regulate DGAT1, emphasizing the complex interplay between lipid metabolism and cellular stress responses.

Consistent with previous findings (49, 50), we observed that knocking down DGAT1 using inhibitors like A922500 or siRNA increases lipotoxicity in the presence of OA, emphasizing DGAT1's protective role in lipid metabolism. Notably, palmitoleic acid does not induce lipotoxic effects, unlike PA, which highlights the importance of fatty acid saturation in cell outcomes.

Both DGAT1 and DGAT2, which reside in the ER, play essential roles in lipid metabolism (51, 52). Following OA treatment, we observed upregulation of DGAT2 in hepatic cells, as reported in (53), and *Caenorhabditis elegans* suggesting DGAT2's protective role against lipotoxicity through increased TAG synthesis, which sequesters excess FFAs and reduces metabolic dysfunction risk. The differential regulation of DGAT2, PLIN2, and PLIN3 in response to PA and OA aligns with the structural differences in LDs observed (54). The PLIN family, including PLIN2 and PLIN3, is crucial for LD biology (55). PLIN2 maintains LD morphology (56, 57), with its knockdown leading to enlarged LDs and increased TAG hydrolysis (58). PLIN3 stabilizes LDs and is vital for intracellular lipid trafficking (59). Recent studies indicated that PLIN3 is associated with micro-droplets (60). The upregulation of PLIN2 during pathological lipid accumulation in the liver suggests a compensatory mechanism to counteract elevated lipid levels and reduce lipotoxicity, especially given that autophagy blocks can exacerbate cellular stress (61, 62). PLIN2 knockdown in mice has been shown to enhance autophagy and protect against severe ER stress-induced hepatosteatosis and apoptosis (62).

When TAG synthesis is impaired, excess FFAs accumulate in the cytoplasm. Cells can redirect FFAs toward membrane phospholipids (63), and inhibiting DGAT1 can reduce TAG flux while increasing phospholipid synthesis (64). While this adaptive mechanism may temporarily alleviate stress from excess FFAs, it could lead to long-term cellular dysfunction due to altered membrane composition. Fully saturated membrane glycerolipids can induce ER stress (63) and the preferential incorporation of PA into phospholipids, we measured, may indeed contribute to ER stress.

Our findings align with those of (65), which show that both PA and OA elevate basal respiration rates, enhancing mitochondrial activity and overall metabolic demand. However, OA is associated with more efficient ATP synthesis, indicating better coupling of respiration to ATP production. In contrast, PA partially uncouples respiration from ATP synthesis, potentially leading to increased energy expenditure, mitochondrial stress, and dysfunction. This difference may stem from the structural properties of the fatty acids; unsaturated fats like OA generally have a more positive impact on mitochondrial functions (66). The uncoupling of respiration from ATP synthesis and the resulting increase in H₂O₂ production may contribute to the lipotoxic effects of PA we observed. Lipotoxicity is linked to oxidative stress in various cellular models, primarily through increased reactive oxygen species (ROS) and diminished antioxidant defenses [ (67, 68, 69, 70, 71). Our results highlight distinct metabolic responses elicited by PA compared to OA, particularly regarding non-mitochondrial oxygen consumption and peroxisomal β-oxidation. The contributions of peroxisomal versus mitochondrial ROS can vary based on cell type, metabolic state, and the specific fatty acids. In some models, oxidative stress induced by peroxisomal H₂O₂ may have more pronounced lipotoxic effects than those caused by mitochondrial ROS (72). Given that peroxisomal β-oxidation influences oxygen consumption and H₂O₂ production (72), we cannot exclude this pathway's role in PA-induced oxidative stress. CPT1 is essential for transporting fatty acids into the mitochondrial matrix. Since CPT1 expression remains constant across groups, this suggests that fatty acid transport for β-oxidation is not the rate-limiting step in this context. The observed activation of PPARα may indicate signaling through alternative pathways that enhance fatty acid oxidation independently of CPT1 levels. Moreover, OA inhibits de novo lipogenesis (73) and malonyl-CoA, the first product of fatty acid synthesis, negatively modulates CPT1. This relationship should be considered when assessing the differing efficiencies of mitochondrial oxidation observed in our experiments.

While FFAs can be toxic when in excess, they are not inherently harmful if properly regulated and adequately oxidized (74). Efficient mitochondrial β-oxidation allows cells to utilize FFAs for energy, preventing detrimental effects associated with lipid overload. Enhancing mitochondrial β-oxidation has been shown to mitigate the lipotoxic effects of PA in various cell models (13, 75). Thus, strategies aimed at increasing β-oxidation (eg, specific dietary interventions, or pharmacological agents) may benefit conditions related to lipotoxicity.

PPARα plays a crucial role in regulating peroxisomal fatty acid oxidation, which is important for lipid metabolism and energy homeostasis. PPARα is activated by various ligands, including fatty acids, with both PA and OA reported as PPARα ligands (76). We observed a modest but significant increase in PPARα expression following PA treatment, with a more pronounced effect from OA. The downregulation of DGAT1 in response to PA suggests this regulation likely occurs independently of direct PPARα control. Under inflammatory conditions, PPARα activation may downregulate specific target genes as part of a broader adaptive response to lipid overload and cellular stress (76). This raises the question of whether a similar mechanism is at play with PA, warranting further investigation. PPARα has been shown to influence cell fate during ER stress and autophagy inhibition (77). Our results indicate that the PPARα agonist GW7647 completely alleviates PA-induced ER stress and autophagy block while upregulating DGAT1 expression. We acknowledge that a limitation of this study is the reliance on HCC cell lines, which do not fully capture the complexities of liver function, especially in clinical contexts. Therefore, future research that integrates both primary hepatocytes and HCC cell lines will provide a more comprehensive understanding of liver biology related to lipotoxicity.

Overall, our data highlight the intricate relationship between fatty acid metabolism, ER stress, autophagy, and cellular health. By prioritizing strategies to enhance fatty acid oxidation while regulating DGAT1 activity, we may mitigate the lipotoxic effects of PA and promote improved metabolic outcomes.

## Data availability

The data generated in the study are included in the article and supplemental data. Raw data and scripts used for analysis are available upon request.

## Supplemental data

This article contains supplemental data.

## Conflict of interests

The authors declare that they have no conflicts of interest with the contents of this article.

## Figures and Tables

**Supplementary Figure S3:**