# Supplementary material for: Lipotoxicity of palmitic acid is associated with DGAT1 downregulation and abolished by PPARα activation in liver cells
Source: J Lipid Res. 2024 Nov 5;65(12):100692. doi: 10.1016/j.jlr.2024.100692 (PMC11648247; doi:10.1016/j.jlr.2024.100692)
Supplement: Supplemental Table S1 [file mmc1.docx]

**Supplementary Table 1. List of primers**

| **Species** | **Gene** | **Accession Number** | **Sequences 5′-3′** |
| --- | --- | --- | --- |
| *H. sapiens* | *GAPDH* | NM_017008 | F: GCATGGCCTTCCGTGTTCCTACC  R: GCCGCCTGCTTCACCACCTTCT |
|  | *RPLP0* | NM_001002.3 | F: TCGACAATGGCAGCATCTAC  R: ATCCGTCTCCACAGACAAGG |
|  | *DGAT1* | NM_012079.5 | F: GACGGATCCTTGAGATGCTG  R: TGAGCCAGATGAGGTGATTG |
|  | *DGAT2* | NM_032564.4 | F: CAAGAAAGGTGGCAGGAGGT  R: GCTGAAGTTGCAGAAGGCAC |
|  | *CPT1* | NM_001031847.2 | F: ACGGCCAACTGCATGTCC  R: CATCCACCCGTGGTAGG |
|  | *PPARα* | NM_001001928.4 | F: CCTAAGGAAACCGTTCTG  R:TACGTTTAGAAGGCCAGGA |
|  | *PLIN and CER6 predesigned primers were obtained from Bio-Rad (more information is available at www.bio-rad.com/PrimePCR)* | | |
| *C. elegans* | *Act-1* | AH000969.2 | F: GAGCGTGGTTACTCTTTCA  R: CAGAGCTTCTCCTTGATGTC |
|  | *Atf-6* | NM­­_077693.7 | F: ATCGTTGCTCCTGCCTAGTG  R: TCAATTGGCCAGTCCCTGTC |
|  | *Daf-2* | AF012437.1 | F: ATCGTCGGATTCTACTGTACTCCC  R: CCGACATCTGACAATATTCATTCTC |
|  | *Dgat-1* | NM_078222.7 | F: TCCGTCCAGGGTGGTAGTG  R: TGAACAAAGAATCTTGCAGACGA |
|  | *Dgat-2* | NM_001269373.3 | F: GCGCTACTTCCGAGACTACTT  R: GGGCCTTATGCCAGGAAACT |
|  | *Daf-16* | AF032112.1 | F: TCAAGACCTCAAAGCCAATCAACTC  R: ACGAGAAAGAAGGAGTAAGAGGAGG |
|  | *Sp-Xbp-1* | NM_001083177.4 | F: TGCCTTTGAATCAGCAGTGG  R: ACCGTCTGCTCCTTCCTCAATG |
|  | *Unsp-Xbp-1* | NM_001083177.4 | F: AGAAGTCGCGGTGAGGTTG  R: CCTGTTCCCACTGCTGAG |
